# Tamoxifen and oxidative stress: an overlooked connection

**DOI:** 10.1007/s12672-021-00411-y

**Published:** 2021-05-27

**Authors:** Nermin S. Ahmed, Marek Samec, Alena Liskova, Peter Kubatka, Luciano Saso

**Affiliations:** 1 Department of Pharmaceutical Chemistry, Faculty of Pharmacy and Biotechnology, German University in Cairo, Cairo, 11835 Egypt; 2 Department of Obstetrics and Gynecology, Jessenius Faculty of Medicine, Comenius University in Bratislava, 03601 Martin, Slovakia; 3 Department of Medical Biology, Department of Experimental Carcinogenesis (Biomedical Center Martin, Division of Oncology), Jessenius Faculty of Medicine, Comenius University in Bratislava, Malá Hora 4, 03601 Martin, Slovak Republic; 4 Department of Physiology and Pharmacology “Vittorio Erspamer”, Sapienza University, P.le Aldo Moro 5, 00185 Rome, Italy

**Keywords:** Apoptosis, ER, Oxidative stress, Resistance, Tamoxifen

## Abstract

Tamoxifen is the gold standard drug for the treatment of breast cancer in pre and post-menopausal women. Its journey from a failing contraceptive to a blockbuster is an example of pharmaceutical innovation challenges. Tamoxifen has a wide range of pharmacological activities; a drug that was initially thought to work via a simple Estrogen receptor (ER) mechanism was proven to mediate its activity through several non-ER mechanisms. Here in we review the previous literature describing ER and non-ER targets of tamoxifen, we highlighted the overlooked connection between tamoxifen, tamoxifen apoptotic effects and oxidative stress.

## Introduction

Tamoxifen **(1)** is a blockbuster drug and a bestseller for hormonal dependent breast cancer. It is the most commonly prescribed drug for estrogen receptor (ER) positive breast cancer patients. For the past 40 years, it succeeded in saving lives. Tamoxifen successful use for treatment and prevention of breast cancer has allowed it to stand the competition with emerging treatments. Tamoxifen cost effectiveness, made it a golden standard treatment for decades [1]. There is a huge interest not only in tamoxifen but also in its hydroxylated metabolites 4-hydroxy tamoxifen (4OH-TAM) **(2)** and endoxifen **(3)**, the study of those active metabolites has helped in understanding the mechanism of action of tamoxifen in breast cancer [2].
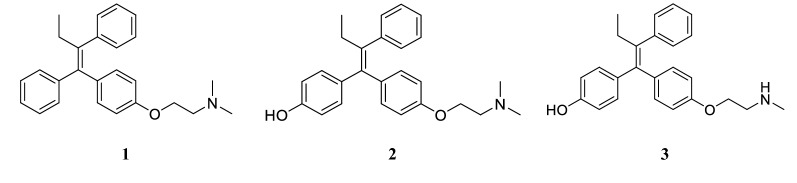


Tamoxifen is pharmacologically classified as Selective Estrogen receptor modulator (SERM). SERMs are a group of compounds bearing diverse chemical scaffolds; SERMs interact with ERs either agonistically or antagonistically based on the target tissue and physiological context [3].

In 1987, Jordan et al*.* discovered that tamoxifen and raloxifene perceived then as ‘’antiestrogen’’ showed estrogenic effects on bones in ovariectomized rats [4]. The discovery of ERβ as another subtype of ER was another reason to revolutionize the idea on nuclear receptor functioning [5].

**Fig. 1:**
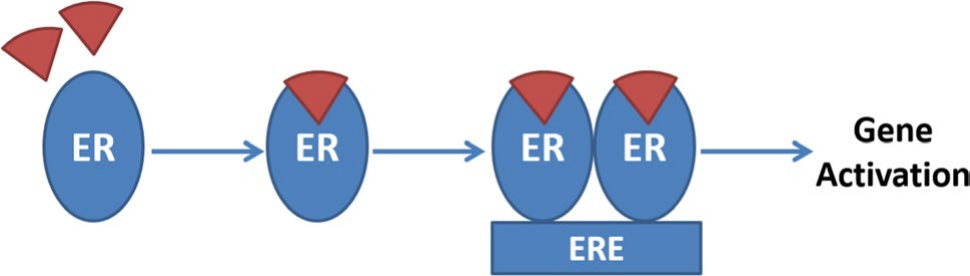
Direct genomic ligand dependent ER mechanism

The oversimplified interpretation of SERM action described by agonist/antagonist was no longer acceptable for nuclear hormone physiology. Therefore, broad insights were needed to understand the molecular basis of ER/SERM actions. An old school interpretation of ER/SERM action is the ligand dependent mechanism, when ligand binds to ER, the heat shock proteins dissociates and the two-receptor subunits dimerize. The receptor dimer then associates with ERE (Estrogen Receptor Element), this in term recruits and modulates the so-called co-regulatory proteins namely co-activators and co-repressors. Co-activators bridges the Estrogen/ER or SERM/ER complex to the basal transcription machinery leading to histone acetylation and gene transcription whereas co-repressor recruitment leads to the exact opposite action (Fig. 1) [6, 7].

One of the major differences between Estrogen/ER complex and SERM/ER complex is the way they recruit co-activators. E2 interact with AF2 domain of ER and this amplifies the gene expression whereas SERM prevents this interaction and its consecutives transcriptional effects [8].

**Fig. 2:**
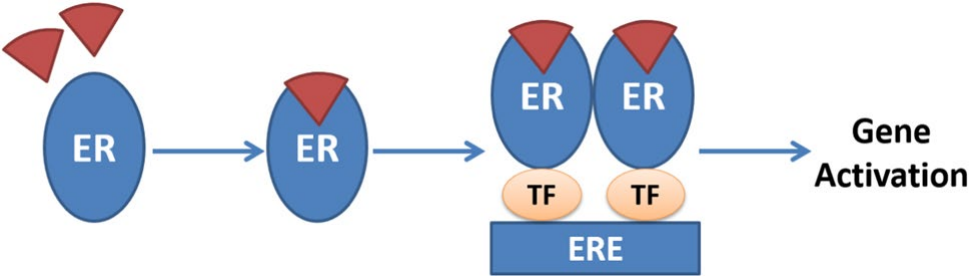
Indirect genomic ligand dependent ER mechanism

In some cases, the promoters of estrogen regulated genes may not possess an ERE sequence, so another indirect genomic ligand dependent mechanism takes over. The ER ligand complex binds to transcription factors such as AP-1 and Sp1 leading to gene activation. This mechanism provides some interpretation to the differential activities of Estrogen/ER complex and SERM/ER complex. It is noticed that while a SERM/ER complex can cause activation to AP-1 sites on ERα, they can cause decreased activation through to AP-1 sites on ERβ (Fig. 2).

**Fig. 3:**
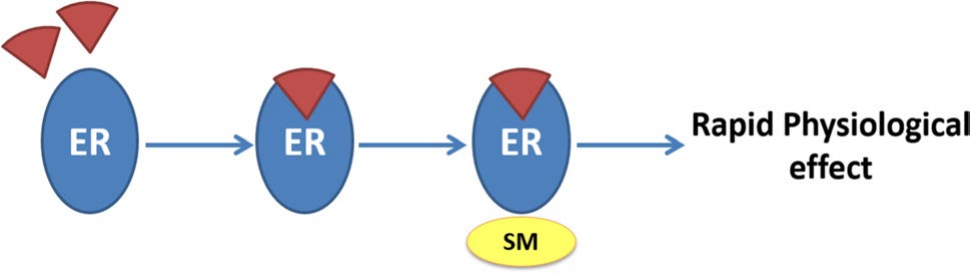
Non-genomic ligand dependent ER mechanism

The ability of ER and SERM to elicit very rapid responses on a wide range of cells and organs is attributed to non-genomic mediated actions. It is postulated that a cell surface ER is probably linked to a G-protein. The non-genomic effects are seen through a cascade of events including secondary messengers [9, 10] (Fig. 3).

**Fig. 4:**
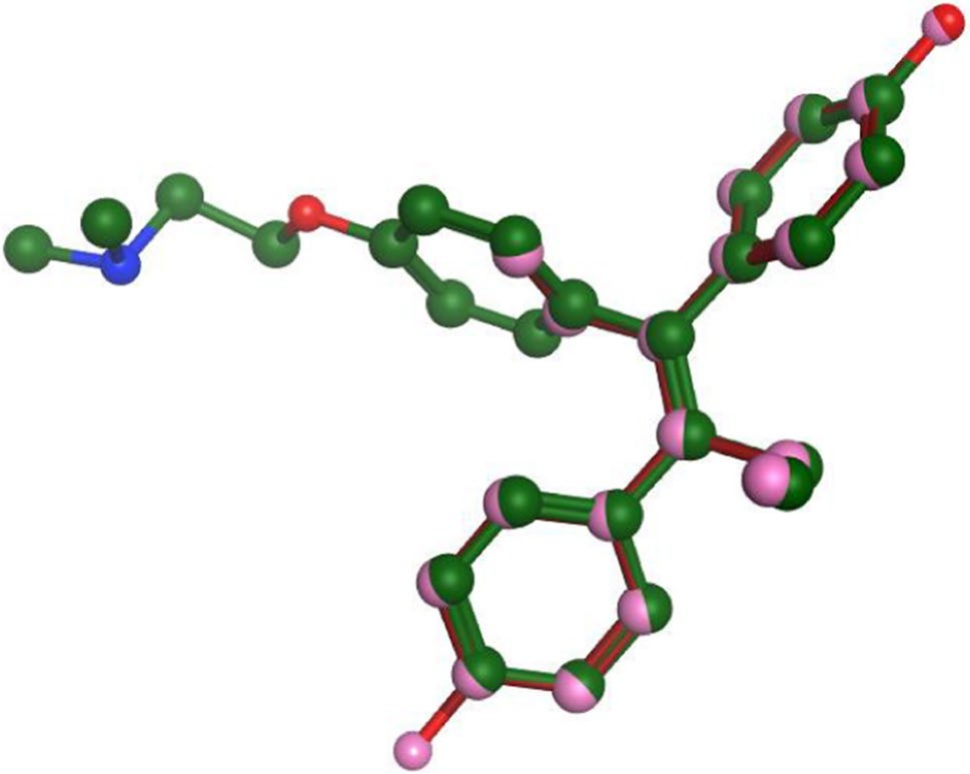
Overlay of 4-OHTAM in (green) with DES (red)

The chemical structure of SERM provide some basic interpretation for their dual pharmacological activity. Medicinal chemists invested a lot in researching the differences in chemical structure between full agonists, full antagonists and SERM. These investigations provide an insight of optimum chemical structures that can be used as ideal SERMs. A basic pharmacophore for all SERMs is reported, at least two aromatic rings are arranged in a stilbene like arrangement [11] and a minimum of one phenolic OH is essential for optimum binding [12]. An overlay between co-crystallized 4-hydroxytamoxifen and diethylstilbestrol (DES) showed that the co-crystallized phenol containing ligand shows an important interaction between the OH group of the ligand, a water molecule and Glu 353/Arg 394 residues [13] (Fig. 4).

**Fig. 5:**
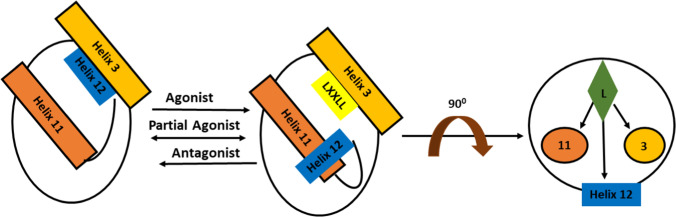
Helix 12 controlling transcription activity of ER

All SERM bear an additional structural feature, a phenyl ring with a bulky substituent at position 4 between the two core phenyls. This protruding substituent moves helix 12 from its agonistic conformation and displaces it from the receptor opening. When the receptor binds to an agonist, helix 12 hydrophobic groove becomes exposed to the nuclear box recognition sequence containing the key **LXXLL** motif. This sequence is common to the many coactivator proteins that are essential for successful ER transcriptional activity. This agonistic mechanism does not occur when a SERM is bound to the ligand binding domain (LBD), as helix 12 dislocates and occupies the space for the coregulatory protein recognition sequence LXXLL binding [14] (Fig. 5).

## Tamoxifen history and development

Tamoxifen was first given the name: compound ICI 46,474 in 1962 by ICI Company. It was first synthesized as a part of a project that aims to developing contraceptive pills. The failure of the compound to elicit anti-estrogenic effect on the ovary and its ability to stimulate ovulation was a great disappointment to ICI pharmaceuticals. Aiming to save the company’s investments, it was suggested to repurpose tamoxifen for breast cancer patients. It received the first approval as a drug for palliative care in late stage breast cancer in 1982. Tamoxifen that started as an orphan drug turned to a bestselling medicine in a journey that started in the eighties. The clinical trials showed its potential use as adjuvant to surgery and chemotherapy in the breast cancer early stages. This was followed by an additional clinical use as a preventive agent in women who are at high risk of developing breast cancer. Tamoxifen was the first chemopreventive agent for any cancer, this extended the market for similar drugs and increased the research focus on chemopreventive agents [15]. By the end of 1970, a relatively small number of breast cancer patients were admitted to clinical trials involving ten weeks treatment with tamoxifen, the tumor regressed in 67% of them in a detectable and clear manner. These findings were similar to results of patients maintained on DES treatment, yet the lack of toxicity and absence of any severe side effects compared to DES boosted the future of tamoxifen [16]. Once tamoxifen was proven effective in both early and late stage breast cancer, many rivals emerged. Tamoxifen yet had two competitive advantages namely lacking androgenic activity and low incidence of side effects.

**Fig. 6:**
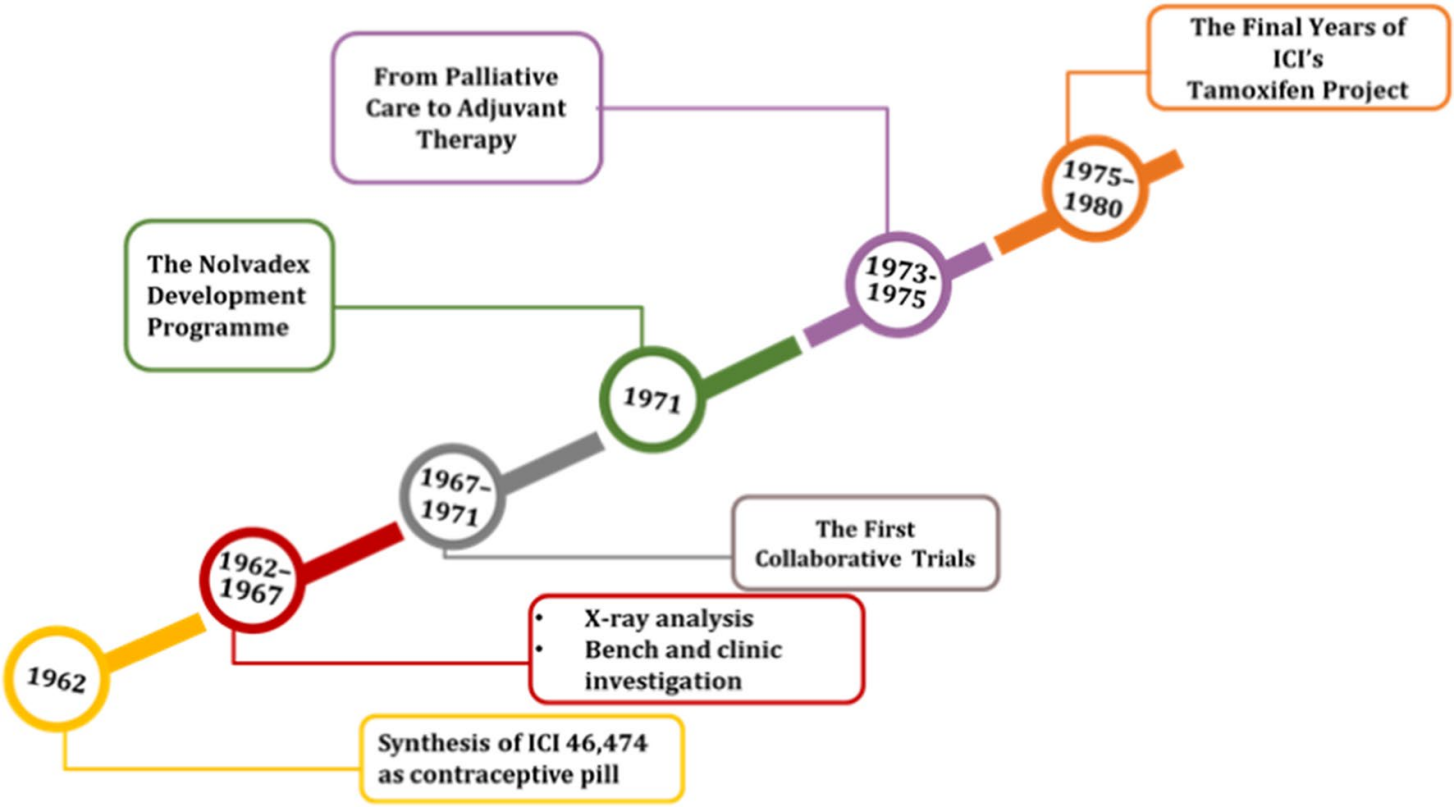
Timeline of tamoxifen development

1975–1980 were the glorious years of tamoxifen discovery project; Clinical trials proved that tamoxifen shows a strong response in old patients with recurrent breast cancer and/or radiotherapy. This action dictated further studies to investigate the ability of tamoxifen or its metabolite to work via non-ER mediated mechanisms. Therefore, tamoxifen was screened in progesterone, androgen and prostaglandin synthetase (PGS) inhibitor screens to find out whether any of these are possible binding targets for tamoxifen [1]. Results showed tamoxifen is a potent inhibitor of PGS that can relieve bone aches in advanced breast cancer patients. This further supported its use in adjuvant chemotherapy and raided the scientific interest in finding new analogues with anti-estrogenic activity and no estrogenic effects and most important with potent PGS inhibitory actions [15]. Clinical data showed that Nolvadex could be used effectively in both pre- and postmenopausal women with ER positive breast cancer tissues. 5–10% of the ER-negative breast cancers have also shown sensitivity to tamoxifen treatment via ER independent signaling pathways [17, 18]. Research findings also highlighted the successful use of tamoxifen in prophylaxis and in remarkably lowering the incidence of recurrence in high-risk patients [19, 20]. The story of tamoxifen from a failing contraceptive to a blockbuster is considered as an inspiring story of pharmaceutical innovation (Fig. 6).

## Tamoxifen non- ER targets

At early times, tamoxifen was believed to work via basic ER competitive antagonistic mechanisms. Nowadays, there are immense knowledge to confirm tamoxifen actions are complex and are not limited to its ER modulator properties. Tamoxifen antitumor effect is mainly attributed to the selective blockage of the ERα expressed in some cancer types yet its indications now go beyond breast cancer to many ER-negative tumors. Many researchers were encouraged to investigate new targets for tamoxifen other than ER. Some of the targets investigated included:

### Protein kinase C (PKC)

One of the initial targets investigated was Protein kinase C (PKC). It plays an essential role in intracellular signaling and cell growth regulation. Tamoxifen inhibited PKC in a manner similar to Ro31-8220, a PKC specific inhibitor. PKC inhibition induced p21(waf1/cip1), Rb dephosphorylation and G1/S phase cell arrest [21]. Tamoxifen cytotoxic effect was demonstrated on prostate cancer, hepatocellular carcinoma [22] and astrocytoma [23]. Tamoxifen can bind directly to PKC epsilon (a PKC isotypes that is characterized as a calcium-independent kinase) in MCF-7 cell culture hindering both differentiation and growth [24].

### Metalloproteinase

Tamoxifen successfully up-regulated expression of metalloproteinases-1, TIMP-1, and down-regulated expression of matrix metalloproteinase 9, MMP-9. Inhibition of proteases inhibited cell invasion in the *in-vitro* assays, tamoxifen inhibited metastasis in both ER-positive like lung adenocarcinoma SPC-A-1 and breast cancer lines MCF-7 [25]. Tamoxifen was also active on ER-negative cell lines like human thyroid cancer cells and B16-BL6 murine melanoma cell lines [26]. The anti-metastatic effect was proven *in- vivo* in murine model where tamoxifen led to a major inhibition of melanoma metastasis in particular to the lungs [27]. Tamoxifen use led to reduction in tumor size and inhibition of metastasis in Fischer rats [28].

### Vascular endothelial growth factor (VEGF) and angiopoietin-1

**Fig. 7:**
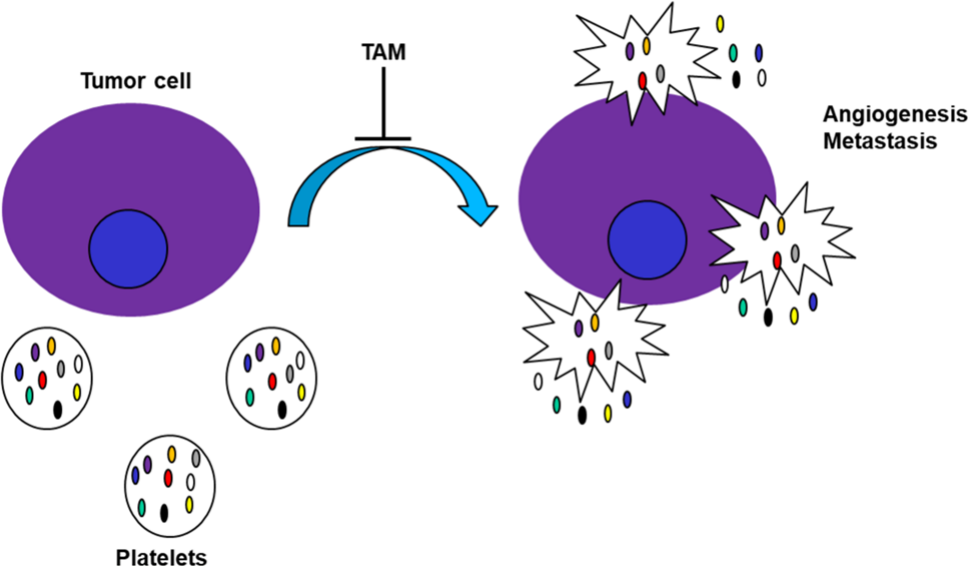
Tamoxifen and 4-OHTAM anti-angiogenic mechanism

Tamoxifen possesses potent anti-angiogenic properties that facilitates its tumor growth and metastasis inhibitory activity in both ER + and ER- cell lines [29, 30]. Tamoxifen and (4-OHTAM) inhibit platelet activation that consecutively lowers the vascular endothelial growth factor (VEGF) and increases the angiopoietin-1. These two proteins are key players in angiogenesis and metastasis [31] (Fig. 7).

In addition to the aforementioned proteins, reports highlighted the involvement of cyclin-dependent endothelial cell growth [29], transforming growth factor beta (TGF-β) [32] and basic fibroblast growth factor (bFGF) in the antiangiogenic action of tamoxifen and its metabolites [33].

### ABC transporter family

Another tamoxifen target are proteins responsible for chemotherapeutic efflux of antitumor agents, this action is responsible for multidrug resistance mechanism in cancer patients (MDR). The proteins involved are transport proteins of the ABC transporter family [34, 35]. Tamoxifen enhanced the activity of some anti-tumor agents via interaction with P-glycoprotein (Pgp).Tamoxifen competes over P-glycoprotein (Pgp) with cytostatic agents, and therefore leads to a recovery of chemotherapeutic effect in particular with cells having an MDR phenotype [36].

**Fig. 8:**
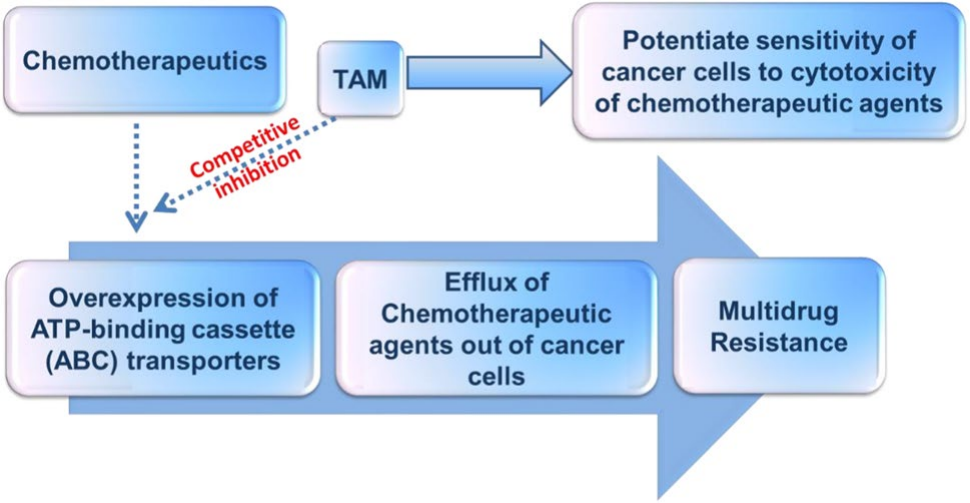
Tamoxifen role in MDR

To further validate Pgp as the potential target, tamoxifen was used to treat monoclonal antibody-Pgp in leukemia cell line K562, it was confirmed that tamoxifen competed with the antibody for the binding [37]. Another target confirmed using the same technique was MRP1 in human cervical carcinoma cell line HeLa [38]. Results ascertain that tamoxifen interacts with major MDR markers Pgp, MRP1 and LRP, and this in turn affects how antitumor drugs bind to transporter proteins, consequently inhibits MDR mechanism [37]. It is worth mentioning that this kind of interaction might lead to a decrease in intracellular concentration of tamoxifen and thereof lower its ER mediated activities. Thus, cells over expressing transporter proteins may show poor prognosis to tamoxifen use. This should not be mistaken as tamoxifen being effluxed out of tumor cells but rather as binding to Pgp, MRP1 or LRP and not to tamoxifen primary target [39] (Fig. 8).

## Tamoxifen resistance and its clinical implication

Tamoxifen outstanding clinical outcomes have been devaluated by the fact that most of the patients with metastasis and about 40% of patients on adjuvant tamoxifen relapse [40]. Relapse occurs due to tamoxifen de novo and acquired resistance; this created a crucial need to understand the mechanism of these types of resistance, this understanding can help developing strategies to overcome tamoxifen resistance.

Tamoxifen resistance has been related to multiple mechanisms, all of which leads to dysregulation of ER. Major mechanisms include modulation of ER signaling, activation of oncogenic signaling pathways such as PI3K/Akt/Mtor and interference with growth factor signaling, and transcription factors [41].

Since most of the actions of tamoxifen are mediated via ER, ER expression has been considered a prognostic marker for tamoxifen effectiveness and therefore loss of ER expression is associated with de novo drug resistance. This is further confirmed by failure of tamoxifen to treat ER/PgR negative tumors [42, 43]. Reports suggested that around 20% of patients may suffer from acquired resistance despite they responded to anastrozole or to fluvestrant suggesting a role for ER in growth regulation even in many tamoxifen-resistant cases [44, 45]. Gene mutations are another common mechanism of tamoxifen resistance, although there has been a number of mutations that are induced in vitro yet only very few of those mutations are observed in patients [46, 47]. Mutations involving Asp351 were confirmed to abolish both agonistic and antagonistic activity of 4-OHTAM [48, 49]. Another distinctive mutation is changing Lys303 to Arg; this mutation was detected in 33% of patients with breast hyperplasia. This mutation promoted co-activator binding even with low estrogen levels and led to disturbance in ER dependent cell growth and consecutively induced drug resistance [50]. Some of the common mutation in LBD residues was not detected in the primary tumors obtained prior to endocrine treatment. These data indicate a novel mechanism of acquired endocrine resistance in breast cancer. Further studies are needed to assess the frequency of such mutations among patients and explore ways to inhibit its activity and restore sensitivity to hormonal therapy [51]. In 2015, Jeselsohn et al. studied recurrent ESR1 mutations in patients with metastatic ER + disease treated by endocrine therapies including aromatase inhibitors. These mutations appeared as a hotspot inside the LBD, this was accompanied by partial resistance to tamoxifen and fluvestrant and increased metastatic capacity [52]. Samples taken from newly diagnosed metastatic and loco-regional recurrence of endocrine-treated breast cancer showed that hotspot ESR1 mutations could emerge after or during adjuvant endocrine therapy including single-agent TAM, as well as during neoadjuvant endocrine treatment of primary tumors. The occurrence of these mutations usually confers a poor prognosis. Studying theses mutations is essential in dictating suitable endocrine therapies [53].

Some epigenetic mutations like hypermethylation of CpG island leads to transcriptional inactivation of the ER gene. Therefore, promoters methylation status has been used as an indication of tamoxifen resistance development [54].

Spalt-like transcription factor 2 (SALL2), a member of the Cys2His2-like fold group (C2H2) zinc finger transcription factor family plays an essential role in cell growth and tumor progression [55, 56]. The loss of SALL2 abolished serum deprivation-induced cell cycle arrest, whereas SALL2 overexpression suppressed cell growth [57]. These results led to the assumption that SALL2 is a tumor suppressor. A contradicting observation led to considering SALL2 as an oncogenic protein. Researchers concluded that SALL2 ability to induce or suppress tumor depends on the protein and cell environment and the dominant signaling pathways. Ye et al. reported that SALL2 was significantly downregulated during tamoxifen therapy, this led to independent ER tumor growth and it conferred ER resistant phenotype in ER + cancer cells. SALL2 activity could be restored using DNA methyltransferase (DNMT), therefore co-administration of tamoxifen and DNMT inhibitors like 5-Aza-20-deoxycytidine (5-Aza-dC), is a rational approach to re-sensitize patients tamoxifen resistant breast cancer patients [58].

Drug-metabolizing enzymes (DMEs) did not receive much attention despite their possible involvement in tamoxifen metabolic drug resistance. Cytochrome P450 enzymes mediate tamoxifen biotransformation, CYP3A4, CYP2B6, CYP2C9, CYP2C19 and CYP2D6 are the essential isoenzymes for this process. The hydroxylated metabolites, 4-OHTAM **(2)** endoxifen **(3)** showed higher growth inhibition potency than the parent drug [59]. Endoxifen, the most potent metabolite is produced via* N*- demethylation of tamoxifen to form *N*-desmethyltamoxifen (NDM) whereas CYP2D6 catalyzed 4-hydroxylation of NDM to form endoxifen. There are more than 300 variants of CYP2D6 reported, some of them interferers prominently with potential clinical outcome and resistance of tamoxifen [2, 60–65]. Some recent work aimed to develop tamoxifen analogues that can bypass CYP2D6 metabolism and use the esterase enzymes to produce the active metabolites to ensure equal clinical outcomes for all patients [66–68].

**Fig. 9:**
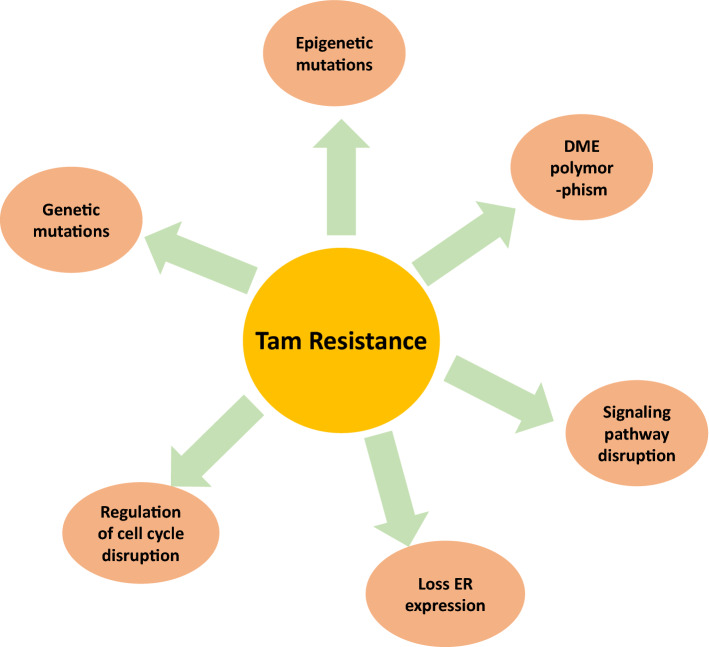
Factors contributing to tamoxifen resistance

The prevalence of CYP2D6*4, CYP2D6*10, CYP3A5*3 and CYP2C19*2 allelic variants and their correlation with tamoxifen resistance was intensively investigated. Studies suggested that lower CYP2D6 activity is associated with diminished clinical results, increased risk of relapse and lowers the chances of free survival. As for CYP3A5 and CYP2C19, there was no significant correlation between CYP3A5*3 and the plasma concentrations of tamoxifen and its metabolites. Confirming with previous studies CYP2C19*2 was associated with better efficacy of tamoxifen. CYP3A5 and CYP2C19 plays a minor role compared to CYP2D6 enzyme [69–74]. Therefore, researchers concluded there is an association between CYP genotypes and clinical pathology of breast cancer. It was noted that there was significant association between intermediate and poor metabolizers of CYP2D6 and CYP2C19 and tamoxifen resistance [75]. The question of whether genetic polymorphisms of CYP2D6 can affect treatment outcome in patients with early postmenopausal breast cancer has been a matter of debate, the necessity of genotyping to guide tamoxifen therapy is even another ongoing argument [74, 76, 77]. Summary of various factors affecting tamoxifen resistance is summarized in Fig. 9.

## Mechanisms of tamoxifen-induced apoptosis

**Fig. 10:**
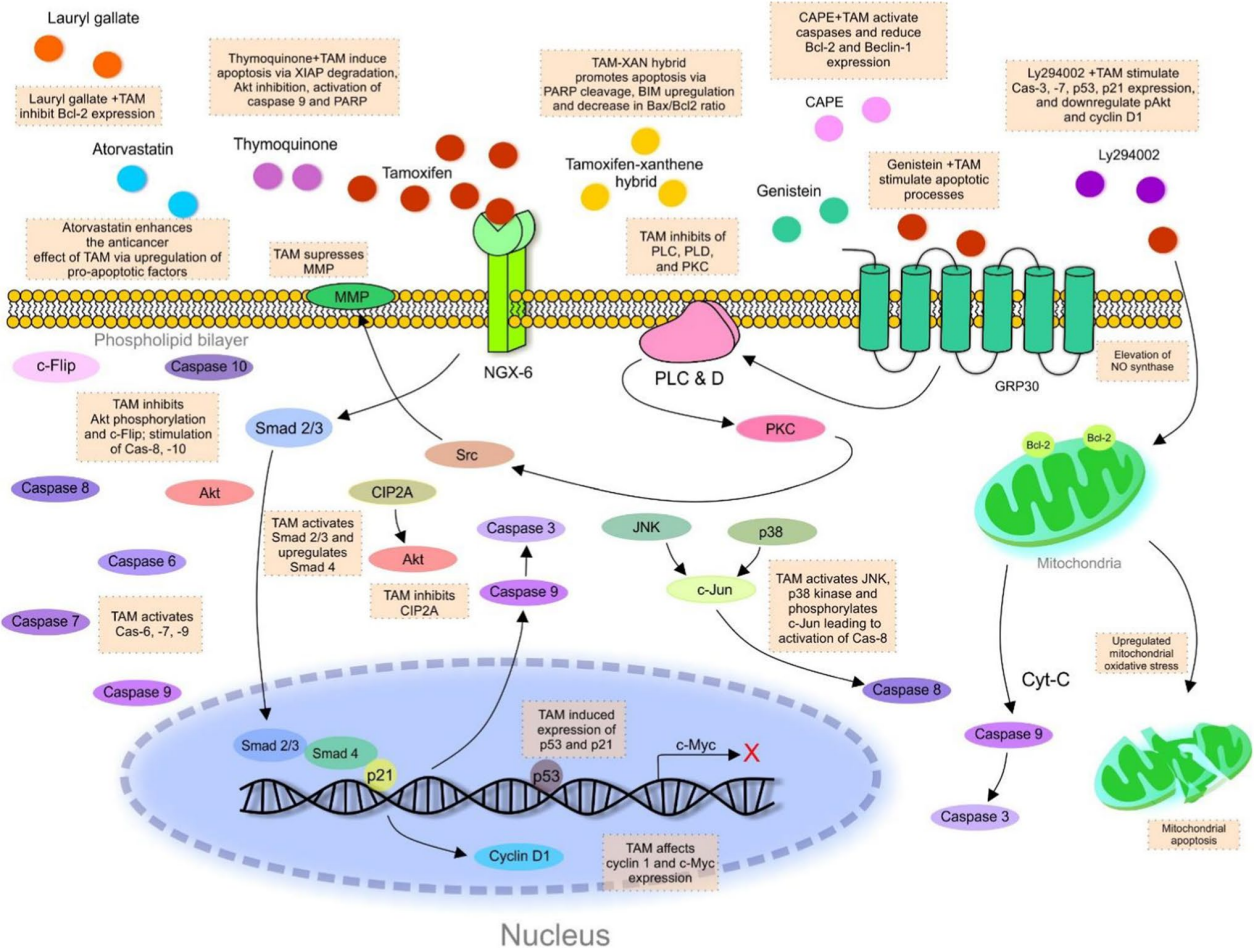
Mechanism of tamoxifen induced apoptosis

Apoptosis plays a vital role in the modulation of neoplastic transformation of cells. Tamoxifen induces apoptosis via non-ER dependent mechanisms including inhibition of phospholipase C-, D- and protein kinase C-mediated pathways [78]. Tamoxifen additionally activates caspases 6, 7 and 9 [79]. Tamoxifen inhibits AKT and JNK activation yet it activates MAP kinase in glioma C6 cells [80]. Tamoxifen induces apoptosis in cells overexpressing Bcl2; it activates c-Jun N-terminal kinase (JNK), p38 kinase and phosphorylation of c-Jun, which in turn activates caspase 8 [81]. This mechanism was strictly limited to cells overexpressing Bcl2. Tamoxifen induced expression of p53 and p21(waf1/cip1), and stimulated G0-G1 phase cell cycle arrest [82]. Tamoxifen significantly affected cyclin D and cMyc expression and stimulated caspases 6, 7 and 9 [83]. Tamoxifen stimulates apoptosis and oxidative stress in a mechanism that involves elevation of mitochondria nitric oxide (NO) synthase. Tamoxifen suppresses mitochondrial respiration and decreases cytochrome c; this in turn stimulates mitochondrial lipid peroxidation. This eventually leads to the induction of tumor apoptosis [84, 85]. It is important to note that tamoxifen regulates intracellular cascade, leading to the suppression of mitochondrial respiration, which stimulates apoptosis. As it has been identified by recent studies, tamoxifen stimulated the activity of caspase-3 in MDA-MB-231 and BT-20 breast cancer cell lines. Besides, tamoxifen demonstrated a pro-apoptotic effect via stimulation of c-Jun NH2-terminal kinase 1 (JNK1) [86]. Tamoxifen inhibited phosphorylation of Akt (pAkt) and c-FLIP in cells isolated from cholangiocarcinoma tumor xenografts. Overexpression of c-FLIP is associated with inhibition of tamoxifen-induced apoptosis, whereas deletion of a calmodulin-binding domain on c-FLIP restored sensitivity to tamoxifen. In vitro, tamoxifen treatment led to stimulation of caspase 8 and 10 in Sk-ChA-1 cholangiocarcinoma cell line. Therefore, the pro-apoptotic effect of tamoxifen is partially dependent on the inhibition of FLIP expression and inhibition of pAkt and stimulation of caspase activity in vivo and in vitro, respectively [87]. Interestingly, cross-connection between the lower level of nasopharyngeal carcinoma-associated gene 6 (NGX6) and cancer cell resistance to tamoxifen has been observed. Tamoxifen activated Smad-2/3 and also upregulated expression of Smad-4 in NGX-6-expressing TRM-7 cells leading to inhibition of proliferation and induction of apoptosis [88]. Furthermore, tamoxifen-induced apoptosis was mediated via cancerous inhibitor of protein phosphatase 2A (CIP2A) /protein phosphatase 2A (PPA2) / phospho-Akt (p-Akt) cascade in MDA-MB-231, MDA-MB-468, MDA-MB-453 and SK-BR-3 cells [89]. In another study, tamoxifen demonstrated anti-cancer potential against MCF-7 breast cancer cell line via induction of apoptosis and inhibition of invasion. Tamoxifen intervention significantly reduced mitochondrial membrane potential (MMP) and the amount of ATP. Notably, the lower level of MMP represents a sign of early cell apoptosis. Moreover, once is the level of MMP decreased, apoptosis is irreversible [90]. The anti-cancer role of tamoxifen via up-regulation of factors associated with apoptosis was observed in MCF-7 cell line. More in-depth molecular analysis revealed numerous genes that were upregulated after incubation with tamoxifen. Most of them were categorized as pro-apoptotic or growth-related intermediates, that contributed to MAPK and/or Tp53 signaling pathways [91]. It is worth mentioning that several natural or synthetic drugs can modulate the pro-apoptotic efficacy of tamoxifen. Recent evidence suggested the beneficial aspect of co-treatment by atorvastatin combined with tamoxifen via elevated expression of pro-apoptotic factors (such as Bax and cytochrome C) in melanoma B16F10 cells compared to groups treated by only with tamoxifen or atorvastatin [92]. Similarly, naturally occurring flavonoids genistein combined with tamoxifen exerted a synergic effect against HepG2 hepatocellular carcinoma cells via stimulation of pro-apoptotic processes [93]. Importantly, TAM combined with Caffeic acid phenethyl ester (CAPE) induced apoptosis through the activation of caspases and induction of DNA fragmentation in MCF-7 cells. Additionally, the synergic effect of TAM and CAPE decreased the level of Bcl-2 and Beclin-1. In this regard, combination of TAM with CAPE may improve cytotoxic efficacy of TAM and overcome possible resistance or lower toxicity [94]. Besides, natural occurring phytochemical thymoquinone increased tamoxifen induced apoptosis in MCF-7 and MDA-MB-231 breast cancer cell lines [95]. Thymoquinone combined with TAM induced apoptosis via X-linked inhibitor of apoptosis protein (XIAP) degradation and Akt inhibition and subsequent activation of caspase-9 and PARP cleavage in breast cancer cells. Also, co-administration of TAM- thymoquinone induced apoptosis through the downregulation of Bcl-xL, Bcl-2 and higher expression of proapoptotic Bax, apoptosis-inducing factor (AIF), p27, and cytoC in breast cancer cells [96]. Moreover, the synergic pro-apoptotic effect of lauryl gallate and tamoxifen was demonstrated in MCF-7 breast cancer cells. The combination of both substances resulted in a lower expression of Bcl-2 [97]. Additionally, co-treatment by tamoxifen and PI3K inhibitor LY294002 synergically induced expression of pro-apoptotic genes such as caspase-3 and -7, and p53 and p21, respectively. Further analysis revealed downregulation of pAkt and cyclin D1 after combined treatment. On the other hand, downregulation of anti-apoptotic genes, including Bcl-2 and survivin, was detected after LY294002 and tamoxifen co-treatment in MCF-7 cells [98]. In a recent study, tamoxifen-xanthene hybrid induced mitochondria-mediated apoptosis due to activation of PARP cleavage, upregulation of Bim gene expression and increase in Bax/Bcl2 ratio in MCF-7 cells [99]. Figure 10 represents an overview of mechanisms associated with tamoxifen-induced apoptosis.

## Tamoxifen-induced toxicity associated with oxidative stress and its prevention

Tamoxifen stimulates the generation of oxidative stress that can result in the damage of cellular structures [100]. Indeed, oxidative stress and inflammation contribute to the occurrence of tamoxifen-induced adverse event, hepatotoxicity [101]. However, current research offers several potential ways to prevent tamoxifen-induced toxicity associated with oxidative stress. Several reports suggested supplementation that exerted protective effects against hepatotoxicity induced by tamoxifen in vivo. Zinc inhibited oxidative stress and improved antioxidant activity through abrogation of tamoxifen-induced decrease of hepatic activity of superoxide dismutase (SOD), catalase (CAT), and glutathione peroxidase (GPx) and increase of malondialdehyde (MDA) level in female Wistar rats [101]. Similarly, pretreatment with thymoquinone, a natural compound found in *Nigella sativa* seeds, normalized SOD activity and inhibited the rise of TNF-α in the model of tamoxifen-induced hepatotoxicity of female rats [102]. The co-treatment with lycopene increased the level of SOD, CAT, GPX, and GSH and downregulated the activity MDA as well [103]. Moreover, the combination of sodium butyrate and tamoxifen upregulated CAT, SOD, and GPx1 genes in rat bone marrow cells. Therefore, such combinatory-treatment could be associated with the modulation of genotoxic effects of tamoxifen by reducing oxidative stress [104]. Besides, as was demonstrated in tamoxifen-treated female Sprague–Dawley rats, tamoxifen-phospholipid complex could ameliorate the hepatotoxicity of tamoxifen by diminishing toxicity markers such as lipid peroxidation or increasing the activity of antioxidant enzymes [105]. In another study, female Wistar rats treated with pure tamoxifen was compared to the control group on 7,12-dimethylbenz(a)anthracene (DMBA) and to a group treated with tamoxifen loaded poly(d,l-lactic acid) (PLA) nanoparticles (NPs), the tumor size was significantly reduced in groups treated with NPs as compared to pure Tmx and untreated group [106]. Also, encapsulation of tamoxifen in solid lipid nanoparticles exerted less adverse effects associated with reduced total oxidant status in a model of ovariectomized female Sprague–Dawley rats [107].

## Oxidative stress implications for tamoxifen therapy and resistance

Mechanisms associated with oxidative stress contribute to tamoxifen-induced hepatotoxicity [101]. However, tamoxifen exerts anti-tumor efficacy independently of ER expression through various mechanisms, such as those associated with oxidative stress [90].

As discussed in previous chapters, the resistance to tamoxifen is mediated through various mechanisms. However, in the context of the interaction of tamoxifen and oxidative damage, the effect of oxidative stress on tamoxifen resistance must also be highlighted. Tumor cells promote the resistance to tamoxifen by elevated levels of ROS-protecting enzymes. Increased levels of anti-oxidant proteins were observed in tamoxifen-resistant MCF-7 cells when compared with non-resistant MCF-7 cells through Nrf2/anti-oxidant response element (ARE) activation [108]. The accumulation of tamoxifen and its metabolites increased oxidative stress in ER + as well as ER- breast cancer cells resulting in cell death. However, as a respond, breast cancer cells increased the expression of Nrf2 that led to the activation of ARE and increased transcription of genes associated with anti-oxidation and multidrug resistance transporters, thus increasing the survival from oxidative damage induced by tamoxifen [109].

Nevertheless, the combinatorial treatment with tamoxifen and antioxidants could promote anti-cancer efficacy of tamoxifen as was demonstrated in the model of DMBA-induced mammary carcinogenesis in Sprague–Dawley rats in which an administration of tamoxifen in combination with riboflavin, niacin, and coenzyme Q10 restored lipid peroxide level and the activity of anti-oxidants accompanied by enhanced antitumor activity [110]. However, antioxidants can potentially decrease the efficacy of anti-cancer therapies. Cancer cells are often characterized by increased accumulation of vitamin C when compared to normal cells and may be therefore better protected against ROS-associated negative effects. The potential detrimental effects of vitamin C supplementation during cancer treatment is supported by the ability of vitamin C to dose-dependently protect cancer cells from tamoxifen-induced lipid peroxidation demonstrated in breast cancer model in vitro (MCF-7 cells) [111].

Based on the above discussion of available evidence, it can be assumed that the implication of the effects of oxidative stress on the efficacy or resistance to tamoxifen is not straightforward due to the effects of different circumstances obtained from available research results. Tumor cells can develop a resistance to tamoxifen as a result of promoted protection against ROS mediated via increased anti-oxidative enzymes. On the contrary, combinatorial treatment of tamoxifen and antioxidants was demonstrated to promote the anti-cancer activity of tamoxifen. However, this finding is not clear, as in another model of breast cancer, the accumulation of vitamin C by tumor cells led to their protection against the effects of tamoxifen. Therefore, we can conclude that the effects of oxidative stress/antioxidants on the efficacy of tamoxifen are not consistent in terms of resistance or efficacy of tamoxifen and require further investigation.

## Conclusion

In conclusion, the consideration of the effects of tamoxifen on both tumor and non-tumor cells through oxidative stress involves the interaction of several mechanisms associated with the therapeutic success, acquired resistance, or toxicity of tamoxifen.

## References

[1] Quirke VM (2017). Tamoxifen from failed contraceptive pill to best-selling breast cancer medicine: a case-study in pharmaceutical innovation. Front Pharmacol.

[2] Schroth W, Goetz MP, Hamann U, Fasching PA, Schmidt M, Winter S (2009). Association between CYP2D6 polymorphisms and outcomes among women with early stage breast cancer treated with tamoxifen. JAMA.

[3] Jordan VC (2004). Selective estrogen receptor modulation. Cancer Cell.

[4] Jordan VC, Phelps E, Lindgren JU (1987). Effects of anti-estrogens on bone in castrated and intact female rats. Breast Cancer Res Treat.

[5] Kuiper GG, Gustafsson JA (1997). The novel estrogen receptor-beta subtype: potential role in the cell- and promoter-specific actions of estrogens and anti-estrogens. FEBS Lett.

[6] Smith CL, Nawaz Z, O’Malley BW (1997). Coactivator and corepressor regulation of the agonist/antagonist activity of the mixed antiestrogen, 4-hydroxytamoxifen. Mol Endocrinol.

[7] Lonard DM, Smith CL (2002). Molecular perspectives on selective estrogen receptor modulators (SERMs): progress in understanding their tissue-specific agonist and antagonist actions. Steroids.

[8] Metzger D, Ali S, Bornert JM, Chambon P (1995). Characterization of the amino-terminal transcriptional activation function of the human estrogen receptor in animal and yeast cells. J Biol Chem.

[9] Aronica SM, Kraus WL, Katzenellenbogen BS (1994). Estrogen action via the cAMP signaling pathway: stimulation of adenylate cyclase and cAMP-regulated gene transcription. Proc Natl Acad Sci USA.

[10] Benten WP, Stephan C, Lieberherr M, Wunderlich F (2001). Estradiol signaling via sequestrable surface receptors. Endocrinology.

[11] Miller CP, Komm BS (2001). Chapter 15. Targeting the estrogen receptor with SERMs.

[12] Minutolo F, Bertini S, Papi C, Carlson KE, Katzenellenbogen JA, Macchia M (2001). Salicylaldoxime moiety as a phenolic “A-Ring” substitute in estrogen receptor ligands. J Med Chem.

[13] Shiau AK, Barstad D, Loria PM, Cheng L, Kushner PJ, Agard DA (1998). The structural basis of estrogen receptor/coactivator recognition and the antagonism of this interaction by tamoxifen. Cell.

[14] Brzozowski AM, Pike AC, Dauter Z, Hubbard RE, Bonn T, Engström O (1997). Molecular basis of agonism and antagonism in the oestrogen receptor. Nature.

[15] Jordan VC (1988). The development of tamoxifen for breast cancer therapy: a tribute to the late Arthur L Walpole. Breast Cancer Res Treat.

[16] Cole MP, Jones CT, Todd ID (1971). A new anti-oestrogenic agent in late breast cancer. An early clinical appraisal of ICI46474. Br J Cancer..

[17] Cuzick J, Powles T, Veronesi U, Forbes J, Edwards R, Ashley S (2003). Overview of the main outcomes in breast-cancer prevention trials. Lancet.

[18] Naugler C (2012). Estrogen receptor testing and 10-year mortality from breast cancer: a model for determining testing strategy. J Pathol Inform.

[19] Mouridsen HT, Rose C, Brincker H, Thorpe SM, Rank F, Fischerman K (1984). Adjuvant systemic therapy in high-risk breast cancer: the Danish Breast Cancer Cooperative Group’s trials of cyclophosphamide or CMF in premenopausal and tamoxifen in postmenopausal patients. Recent Results Cancer Res.

[20] Mouridsen HT, Rose C, Overgaard M, Dombernowsky P, Panduro J, Thorpe S (1988). Adjuvant treatment of postmenopausal patients with high risk primary breast cancer. Results from the Danish adjuvant trials DBCG 77 C and DBCG 82 C. Acta Oncol..

[21] Rohlff C, Blagosklonny MV, Kyle E, Kesari A, Kim IY, Zelner DJ (1998). Prostate cancer cell growth inhibition by tamoxifen is associated with inhibition of protein kinase C and induction of p21(waf1/cip1). Prostate.

[22] Cheng AL, Chuang SE, Fine RL, Yeh KH, Liao CM, Lay JD (1998). Inhibition of the membrane translocation and activation of protein kinase C, and potentiation of doxorubicin-induced apoptosis of hepatocellular carcinoma cells by tamoxifen. Biochem Pharmacol.

[23] Sharif TR, Sharif M (1998). A novel approach for examining the anti-proliferative effect of protein kinase C inhibitors against human astrocytoma cells. Int J Oncol.

[24] Lavie Y, Zhang ZC, Cao HT, Han TY, Jones RC, Liu YY (1998). Tamoxifen induces selective membrane association of protein kinase C epsilon in MCF-7 human breast cancer cells. Int J Cancer.

[25] Wang X-Y, Wang Y, Liu H-C (2011). Tamoxifen lowers the MMP-9/TIMP-1 ratio and inhibits the invasion capacity of ER-positive non-small cell lung cancer cells. Biomed Pharmacother.

[26] Hoelting T, Siperstein AE, Duh QY, Clark OH (1995). Tamoxifen inhibits growth, migration, and invasion of human follicular and papillary thyroid cancer cells in vitro and in vivo. J Clin Endocrinol Metab.

[27] Matsuoka H, Tsubaki M, Yamazoe Y, Ogaki M, Satou T, Itoh T (2009). Tamoxifen inhibits tumor cell invasion and metastasis in mouse melanoma through suppression of PKC/MEK/ERK and PKC/PI3K/Akt pathways. Exp Cell Res.

[28] Xing RH, Mazar A, Henkin J, Rabbani SA (1997). Prevention of breast cancer growth, invasion, and metastasis by antiestrogen tamoxifen alone or in combination with urokinase inhibitor B-428. Cancer Res.

[29] Tong S, Chen Q, Shan S-Q, Dewhirst MW, Yuan F (2006). Quantitative comparison of the inhibitory effects of GW5638 and tamoxifen on angiogenesis in the cornea pocket assay. Angiogenesis.

[30] Cáceres W, González S (2003). Angiogenesis and cancer: recent advances. P R Health Sci J.

[31] Davizon-Castillo P, Di Paola J (2017). Tamoxifen suppresses platelet activation-supported angiogenesis and metastasis. Arterioscler Thromb Vasc Biol.

[32] Butta A, MacLennan K, Flanders KC, Sacks NP, Smith I, McKinna A (1992). Induction of transforming growth factor beta 1 in human breast cancer in vivo following tamoxifen treatment. Cancer Res.

[33] McNamara DA, Harmey J, Wang JH, Kay E, Walsh TN, Bouchier-Hayes DJ (2001). Tamoxifen inhibits endothelial cell proliferation and attenuates VEGF-mediated angiogenesis and migration in vivo. Eur J Surg Oncol.

[34] Shen L-Z, Hua Y-B, Yu X-M, Xu Q, Chen T, Wang J-H (2005). Tamoxifen can reverse multidrug resistance of colorectal carcinoma in vivo. World J Gastroenterol.

[35] Hotta T, Tanimura H, Yamaue H, Iwahashi M, Tani M, Tsunoda T (1996). Tamoxifen circumvents the multidrug resistance in fresh human gastrointestinal cancer cells. J Surg Res.

[36] Liu Z-H, Ma Y-L, He Y-P, Zhang P, Zhou Y-K, Qin H (2011). Tamoxifen reverses the multi-drug-resistance of an established human cholangiocarcinoma cell line in combined chemotherapeutics. Mol Biol Rep.

[37] Bogush EA, Ravcheeva AB, Bogush TA, Zabotina TN, Kadagidze ZG, Davydov MI (2007). A new marker of tamoxifen resistance of estrogen receptor-positive breast cancer. Dokl Biochem Biophys.

[38] Bogush TA, Dudko EA, Beme AA, Bogush EA, Kim AI, Polotsky BE (2010). Estrogen receptors, antiestrogens, and non-small cell lung cancer. Biochemistry (Mosc).

[39] Bogush T, Dudko E, Bogush E, Polotsky B, Tjulandin S, Davydov M (2012). Tamoxifen non-estrogen receptor mediated molecular targets. Oncol Rev.

[40] Chang M (2012). Tamoxifen resistance in breast cancer. Biomol Ther (Seoul).

[41] Tryfonidis K, Zardavas D, Katzenellenbogen BS, Piccart M (2016). Endocrine treatment in breast cancer: cure, resistance and beyond. Cancer Treat Rev.

[42] Jaiyesimi IA, Buzdar AU, Decker DA, Hortobagyi GN (1995). Use of tamoxifen for breast cancer: twenty-eight years later. J Clin Oncol.

[43] Ingle JN, Mailliard JA, Schaid DJ, Krook JE, Gesme DH, Windschitl HE (1991). A double-blind trial of tamoxifen plus prednisolone versus tamoxifen plus placebo in postmenopausal women with metastatic breast cancer. A collaborative trial of the North Central Cancer Treatment Group and Mayo Clinic. Cancer.

[44] Osborne CK, Pippen J, Jones SE, Parker LM, Ellis M, Come S (2002). Double-blind, randomized trial comparing the efficacy and tolerability of fulvestrant versus anastrozole in postmenopausal women with advanced breast cancer progressing on prior endocrine therapy: results of a North American trial. J Clin Oncol.

[45] Howell A, Robertson JFR, Quaresma Albano J, Aschermannova A, Mauriac L, Kleeberg UR (2002). Fulvestrant, formerly ICI 182,780, is as effective as anastrozole in postmenopausal women with advanced breast cancer progressing after prior endocrine treatment. J Clin Oncol.

[46] Roodi N, Bailey LR, Kao WY, Verrier CS, Yee CJ, Dupont WD (1995). Estrogen receptor gene analysis in estrogen receptor-positive and receptor-negative primary breast cancer. J Natl Cancer Inst.

[47] Karnik PS, Kulkarni S, Liu XP, Budd GT, Bukowski RM (1994). Estrogen receptor mutations in tamoxifen-resistant breast cancer. Cancer Res.

[48] Wolf DM, Jordan VC (1994). The estrogen receptor from a tamoxifen stimulated MCF-7 tumor variant contains a point mutation in the ligand binding domain. Breast Cancer Res Treat.

[49] MacGregor Schafer J, Liu H, Bentrem DJ, Zapf JW, Jordan VC (2000). Allosteric silencing of activating function 1 in the 4-hydroxytamoxifen estrogen receptor complex is induced by substituting glycine for aspartate at amino acid 351. Cancer Res.

[50] Fuqua SA, Wiltschke C, Zhang QX, Borg A, Castles CG, Friedrichs WE (2000). A hypersensitive estrogen receptor-alpha mutation in premalignant breast lesions. Cancer Res.

[51] Merenbakh-Lamin K, Ben-Baruch N, Yeheskel A, Dvir A, Soussan-Gutman L, Jeselsohn R (2013). D538G mutation in estrogen receptor-α: A novel mechanism for acquired endocrine resistance in breast cancer. Cancer Res.

[52] Jeselsohn R, Buchwalter G, De Angelis C, Brown M, Schiff R (2015). ESR1 mutations—a mechanism for acquired endocrine resistance in breast cancer. Nat Rev Clin Oncol.

[53] Zundelevich A, Dadiani M, Kahana-Edwin S, Itay A, Sella T, Gadot M (2020). ESR1 mutations are frequent in newly diagnosed metastatic and loco-regional recurrence of endocrine-treated breast cancer and carry worse prognosis. Breast Cancer Res.

[54] Ottaviano YL, Issa JP, Parl FF, Smith HS, Baylin SB, Davidson NE (1994). Methylation of the estrogen receptor gene CpG island marks loss of estrogen receptor expression in human breast cancer cells. Cancer Res.

[55] de Celis JF, Barrio R (2009). Regulation and function of Spalt proteins during animal development. Int J Dev Biol.

[56] Kelberman D, Islam L, Lakowski J, Bacchelli C, Chanudet E, Lescai F (2014). Mutation of SALL2 causes recessive ocular coloboma in humans and mice. Hum Mol Genet.

[57] Liu H, Adler AS, Segal E, Chang HY (2007). A transcriptional program mediating entry into cellular quiescence. PLoS Genet.

[58] Ye L, Lin C, Wang X, Li Q, Li Y, Wang M (2019). Epigenetic silencing of SALL2 confers tamoxifen resistance in breast cancer. EMBO Mol Med.

[59] Lim YC, Desta Z, Flockhart DA, Skaar TC (2005). Endoxifen (4-hydroxy-N-desmethyl-tamoxifen) has anti-estrogenic effects in breast cancer cells with potency similar to 4-hydroxy-tamoxifen. Cancer Chemother Pharmacol.

[60] Dehal SS, Kupfer D (1997). CYP2D6 catalyzes tamoxifen 4-hydroxylation in human liver. Cancer Res.

[61] Brauch H, Schwab M (2014). Prediction of tamoxifen outcome by genetic variation of CYP2D6 in post-menopausal women with early breast cancer. Br J Clin Pharmacol.

[62] Ramón y Cajal T, Altés A, Paré L, del Rio E, Alonso C, Barnadas A, et al. Impact of CYP2D6 polymorphisms in tamoxifen adjuvant breast cancer treatment. Breast Cancer Res Treat. 2010;119: 33–38. Doi: 10.1007/s10549-009-0328-y 10.1007/s10549-009-0328-y 19189210

[63] de Souza JA, Olopade OI (2011). CYP2D6 genotyping and tamoxifen: an unfinished story in the quest for personalized medicine. Semin Oncol.

[64] Brauch H, Schroth W, Goetz MP, Mürdter TE, Winter S, Ingle JN (2013). Tamoxifen use in postmenopausal breast cancer: CYP2D6 matters. J Clin Oncol.

[65] Gao L, Sun X, Tu Y, Ågren H, Eriksson LA (2013). Modification of the anticancer drug tamoxifen to avoid CYP2D6 polymorphism. Can J Chem.

[66] Elghazawy NH, Engel M, Hartmann RW, Hamed MM, Ahmed NS, Abadi AH (2016). Design and synthesis of novel flexible ester-containing analogs of tamoxifen and their evaluation as anticancer agents. Future Med Chem.

[67] Ahmed NS, Wober J (2020). Synthesis of novel flexible tamoxifen analogues to overcome CYP2D6 polymorphism and their biological evaluation on MCF-7 cell line. Drug Dev Res.

[68] Ahmed NS, Elghazawy NH, ElHady AK, Engel M, Hartmann RW, Abadi AH (2016). Design and synthesis of novel tamoxifen analogues that avoid CYP2D6 metabolism. Eur J Med Chem.

[69] Sanchez Spitman AB, Moes DJAR, Gelderblom H, Dezentje VO, Swen JJ, Guchelaar HJ (2017). Effect of CYP3A4*22, CYP3A5*3, and CYP3A combined genotypes on tamoxifen metabolism. Eur J Clin Pharmacol.

[70] Wegman P, Elingarami S, Carstensen J, Stål O, Nordenskjöld B, Wingren S (2007). Genetic variants of CYP3A5, CYP2D6, SULT1A1, UGT2B15 and tamoxifen response in postmenopausal patients with breast cancer. Breast Cancer Res.

[71] Charoenchokthavee W, Areepium N, Panomvana D, Sriuranpong V (2017). Effects of CYP2D6 and CYP3A5 polymorphisms on tamoxifen and its metabolites in Thai breast cancer patients. BCTT.

[72] Tucker AN, Tkaczuk KA, Lewis LM, Tomic D, Lim CK, Flaws JA (2005). Polymorphisms in cytochrome P4503A5 (CYP3A5) may be associated with race and tumor characteristics, but not metabolism and side effects of tamoxifen in breast cancer patients. Cancer Lett.

[73] Schroth W, Antoniadou L, Fritz P, Schwab M, Muerdter T, Zanger UM (2007). Breast cancer treatment outcome with adjuvant tamoxifen relative to patient CYP2D6 and CYP2C19 genotypes. J Clin Oncol.

[74] Sanchez-Spitman AB, Swen JJ, Dezentjé VO, Moes DJAR, Gelderblom H, Guchelaar HJ (2021). Effect of CYP2C19 genotypes on tamoxifen metabolism and early-breast cancer relapse. Sci Rep.

[75] Goetz MP, Sangkuhl K, Guchelaar H-J, Schwab M, Province M, Whirl-Carrillo M (2018). Clinical pharmacogenetics implementation consortium (CPIC) guideline for CYP2D6 and tamoxifen therapy. Clin Pharmacol Ther.

[76] Mokbel K, Mokbel K (2017). Does CYP2D6 Genotyping have a Role in Guiding Tamoxifen Therapy?. Biochem Mol Biol J..

[77] Beverage JN, Sissung TM, Sion AM, Danesi R, Figg WD (2007). CYP2D6 polymorphisms and the impact on tamoxifen therapy. J Pharm Sci.

[78] Ahn S-J, Yoon M-S, Hyuk S, Han W, Yoon Y-D, Han J-S (2003). Phospholipase C-protein kinase C mediated phospholipase D activation pathway is involved in tamoxifen induced apoptosis. J Cell Biochem.

[79] Thiantanawat A, Long BJ, Brodie AM (2003). Signaling pathways of apoptosis activated by aromatase inhibitors and antiestrogens. Cancer Res.

[80] Li C, Zhou C, Wang S, Feng Y, Lin W, Lin S (2011). Sensitization of glioma cells to tamoxifen-induced apoptosis by Pl3-kinase inhibitor through the GSK-3β/β-catenin signaling pathway. PLoS ONE.

[81] Moodbidri MS, Shirsat NV (2005). Activated JNK brings about accelerated apoptosis of Bcl-2-overexpressing C6 glioma cells on treatment with tamoxifen. J Neurochem.

[82] Zhang GJ, Kimijima I, Onda M, Kanno M, Sato H, Watanabe T (1999). Tamoxifen-induced apoptosis in breast cancer cells relates to down-regulation of bcl-2, but not bax and bcl-X(L), without alteration of p53 protein levels. Clin Cancer Res.

[83] Han P, Kang J-H, Li H-L, Hu S-X, Lian H-H, Qiu P-P (2009). Antiproliferation and apoptosis induced by tamoxifen in human bile duct carcinoma QBC939 cells via upregulated p53 expression. Biochem Biophys Res Commun.

[84] Nagahara Y, Shiina I, Nakata K, Sasaki A, Miyamoto T, Ikekita M (2008). Induction of mitochondria-involved apoptosis in estrogen receptor-negative cells by a novel tamoxifen derivative, ridaifen-B. Cancer Sci.

[85] Nazarewicz RR, Zenebe WJ, Parihar A, Larson SK, Alidema E, Choi J (2007). Tamoxifen induces oxidative stress and mitochondrial apoptosis via stimulating mitochondrial nitric oxide synthase. Cancer Res.

[86] Mandlekar S, Yu R, Tan TH, Kong AN (2000). Activation of caspase-3 and c-Jun NH2-terminal kinase-1 signaling pathways in tamoxifen-induced apoptosis of human breast cancer cells. Cancer Res.

[87] Pawar P, Ma L, Byon CH, Liu H, Ahn E-Y, Jhala N (2009). Molecular mechanisms of tamoxifen therapy for cholangiocarcinoma: role of calmodulin. Clin Cancer Res.

[88] Zhao W-J, Wang K (2013). NGX6 expression improves the sensitivity of tamoxifen-resistant MCF-7 cells through modulation of the Smad signaling pathway. Int J Oncol.

[89] Liu C-Y, Hung M-H, Wang D-S, Chu P-Y, Su J-C, Teng T-H (2014). Tamoxifen induces apoptosis through cancerous inhibitor of protein phosphatase 2A-dependent phospho-Akt inactivation in estrogen receptor-negative human breast cancer cells. Breast Cancer Res.

[90] Li W, Shi X, Xu Y, Wan J, Wei S, Zhu R (2017). Tamoxifen promotes apoptosis and inhibits invasion in estrogen-positive breast cancer MCF-7 cells. Mol Med Rep.

[91] Rouhimoghadam M, Safarian S, Carroll JS, Sheibani N, Bidkhori G (2018). Tamoxifen-induced apoptosis of MCF-7 cells via GPR30/PI3K/MAPKs interactions: verification by ODE modeling and RNA sequencing. Front Physiol.

[92] Ghasemi M, Malek M, Javanmard SH, Ghasemi A, Esfahani HN, Vaseghi G (2019). Atorvastatin enhances apoptotic effects of tamoxifen on melanoma cancer cells. Bratisl Lek Listy.

[93] Sanaei M, Kavoosi F, Atashpour S, Haghighat S (2017). Effects of genistein and synergistic action in combination with tamoxifen on the HepG2 human hepatocellular carcinoma cell line. Asian Pac J Cancer Prev.

[94] Motawi TK, Abdelazim SA, Darwish HA, Elbaz EM, Shouman SA (2016). Modulation of tamoxifen cytotoxicity by caffeic acid phenethyl ester in MCF-7 breast cancer cells. Oxid Med Cell Longev.

[95] Ganji-Harsini S, Khazaei M, Rashidi Z, Ghanbari A (2016). Thymoquinone could increase the efficacy of tamoxifen induced apoptosis in human breast cancer cells: an in vitro study. Cell J.

[96] Rajput S, Kumar BNP, Sarkar S, Das S, Azab B, Santhekadur PK (2013). Targeted apoptotic effects of thymoquinone and tamoxifen on XIAP mediated Akt regulation in breast cancer. PLoS ONE.

[97] Ghatreh Samani K, Farrokhi E, Tabatabaee A, Jalilian N, Jafari M (2020). Synergistic effects of lauryl gallate and tamoxifen on human breast cancer cell. Iran J Public Health.

[98] Abdallah ME, El-Readi MZ, Althubiti MA, Almaimani RA, Ismail AM, Idris S (2020). Tamoxifen and the PI3K inhibitor: LY294002 synergistically induce apoptosis and cell cycle arrest in breast cancer MCF-7 cells. Molecules.

[99] Catanzaro E, Seghetti F, Calcabrini C, Rampa A, Gobbi S, Sestili P (2019). Identification of a new tamoxifen-xanthene hybrid as pro-apoptotic anticancer agent. Bioorg Chem.

[100] Schieber M, Chandel NS (2014). ROS function in redox signaling and oxidative stress. Curr Biol.

[101] Famurewa AC, Ekeleme-Egedigwe CA, David EE, Eleazu CO, Folawiyo AM, Obasi NA (2020). Zinc abrogates anticancer drug tamoxifen-induced hepatotoxicity by suppressing redox imbalance, NO/iNOS/NF-ĸB signaling, and caspase-3-dependent apoptosis in female rats. Toxicol Mech Methods.

[102] Suddek GM (2014). Protective role of thymoquinone against liver damage induced by tamoxifen in female rats. Can J Physiol Pharmacol.

[103] Adikwu E, Ebinyo NC, Benalayefa O (2020). Protective effect of lycopene against tamoxifen-induced hepatotoxicity in albino rats. Biomed Biotechnol Res J (BBRJ)..

[104] El-Shorbagy HM (2017). Potential anti-genotoxic effect of sodium butyrate to modulate induction of DNA damage by tamoxifen citrate in rat bone marrow cells. Cytotechnology.

[105] Jena SK, Suresh S, Sangamwar AT (2015). Modulation of tamoxifen-induced hepatotoxicity by tamoxifen-phospholipid complex. J Pharm Pharmacol.

[106] Pandey SK, Ghosh S, Maiti P, Haldar C (2015). Therapeutic efficacy and toxicity of tamoxifen loaded PLA nanoparticles for breast cancer. Int J Biol Macromol.

[107] Javid S, Ziamajidi N, Foroughi S, Abbasalipourkabir R (2017). Effects of tamoxifen-loaded solid lipid nanoparticles on the estrogen receptor-α (ER-α) and vascular endothelial growth factor-A (VEGF-A) genes expression in the endometrial tissue of ovariectomized female Sprague-Dawley rats. Int J Biol Macromol.

[108] Kim SK, Yang JW, Kim MR, Roh SH, Kim HG, Lee KY (2008). Increased expression of Nrf2/ARE-dependent anti-oxidant proteins in tamoxifen-resistant breast cancer cells. Free Radic Biol Med.

[109] Bekele RT, Venkatraman G, Liu R-Z, Tang X, Mi S, Benesch MGK (2016). Oxidative stress contributes to the tamoxifen-induced killing of breast cancer cells: implications for tamoxifen therapy and resistance. Sci Rep.

[110] Perumal SS, Shanthi P, Sachdanandam P (2005). Augmented efficacy of tamoxifen in rat breast tumorigenesis when gavaged along with riboflavin, niacin, and CoQ10: effects on lipid peroxidation and antioxidants in mitochondria. Chem Biol Interact.

[111] Subramani T, Yeap SK, Ho WY, Ho CL, Omar AR, Aziz SA (2014). Vitamin C suppresses cell death in MCF-7 human breast cancer cells induced by tamoxifen. J Cell Mol Med.

